# High-resolution vector microwave magnetometry based on solid-state spins in diamond

**DOI:** 10.1038/ncomms7631

**Published:** 2015-03-23

**Authors:** Pengfei Wang, Zhenheng Yuan, Pu Huang, Xing Rong, Mengqi Wang, Xiangkun Xu, Changkui Duan, Chenyong Ju, Fazhan Shi, Jiangfeng Du

**Affiliations:** 1 Hefei National Laboratory for Physics Sciences at the Microsacle and Department of Modern Physics, University of Science and Technology of China, Hefei 230026, China; 2 Synergetic Innovation Centre of Quantum Information and Quantum Physics, University of Science and Technology of China, Hefei, Anhui 230026, China

## Abstract

The measurement of the microwave field is crucial for many developments in microwave technology and related applications. However, measuring microwave fields with high sensitivity and spatial resolution under ambient conditions remains elusive. In this work, we propose and experimentally demonstrate a scheme to measure both the strength and orientation of the microwave magnetic field by utilizing the quantum coherent dynamics of nitrogen vacancy centres in diamond. An angular resolution of 5.7 mrad and a sensitivity of 1.0 μT Hz^−1/2^ are achieved at a microwave frequency of 2.6000 GHz, and the microwave magnetic field vectors generated by a copper wire are precisely reconstructed. The solid-state microwave magnetometry with high resolution and wide frequency range that can work under ambient conditions proposed here enables unique potential applications over other state-of-art microwave magnetometry.

## Abstract (web summary)

Microwave technology is crucial for communications and high-speed electronics. Wang *et al*. now use nitrogen-vacancy defects in diamond to measure the strength and orientation of the magnetic component of a microwave electromagnetic field on the nanoscale.

## Graphical abstract

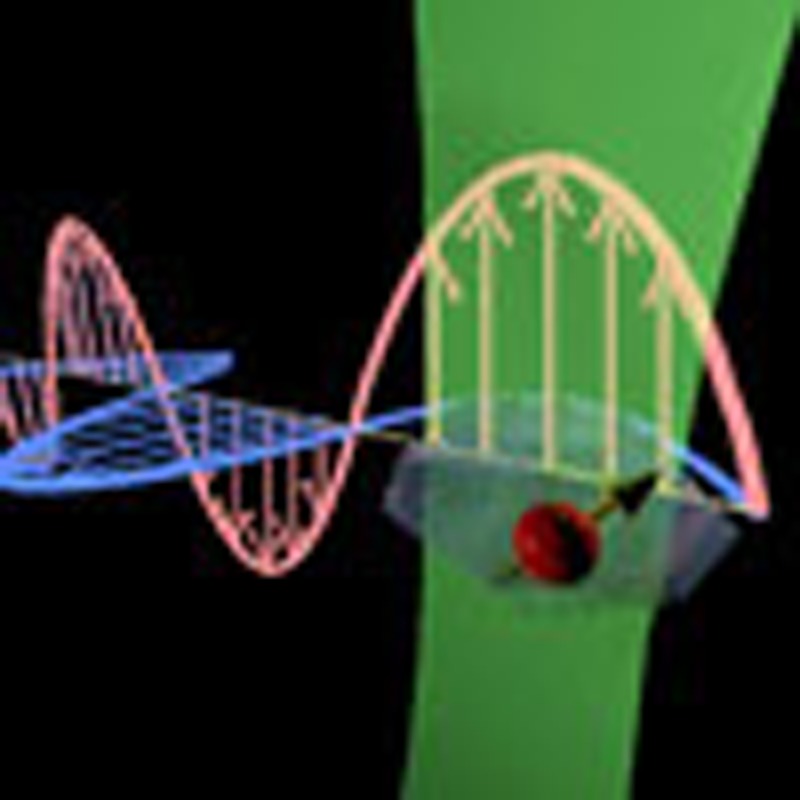

Recent advantages in microwave (MW) technology have led to tremendous developments in communications, high-speed electronics and magnetic resonance. Most of these developments rely on the discovery of nanoscale, high-frequency MW processes^1^,^2^,^3^ and materials^4^,^5^,^6^. Measurement of MW electromagnetic field with high spatial resolution is crucial but has been a long-standing problem. Measurements with high sensitivity and angular resolution have been achieved for MW electric field^7^,^8^. It is not so for the measurement of MW magnetic field vector, despite its importance, for example, in MW antenna test^9^ and the study of the MW response of materials to the magnetic field^5^. Although various approaches, such as coplanar-waveguide-type probe^10^,^11^,^12^,^13^,^14^, superconducting quantum interference devices^15^, cold atoms^16^,^17^ or spin hall effect films^18^, have been applied to measure the MW magnetic field, the sensor size, the extreme measurement conditions required or the lack of vector measurement ability strongly limit their applications.

In this work, we demonstrate a high-resolution vectorial MW magnetometry based on nitrogen vacancy (NV) centres in diamond. The NV centre is a defect that consists of a substitutional nitrogen atom and an adjacent vacancy in the carbon lattice of diamond. The ground spin triplet state of negatively charged NV centres can be initialized and measured by laser illumination and fluorescence intensity under laser illumination, respectively. Owing to its good spin properties, the NV centre has been shown to be a perfect solid-state quantum system for sensing magnetic fields with nanoscale resolution and high sensitivity under ambient conditions^19^,^20^. In previous works, NV centres have been utilized in the measurement of oscillating magnetic fields with frequencies ranging from kilohertz^19^ to megahertz^21^,^22^. Here we propose and then demonstrate experimentally a different scheme based on NV centres to realize the measurement of the vector of the MW magnetic field. The angular resolution and sensitivity are then analysed.

## Results

### Principles of vector MW magnetometry

**Figure 1:**
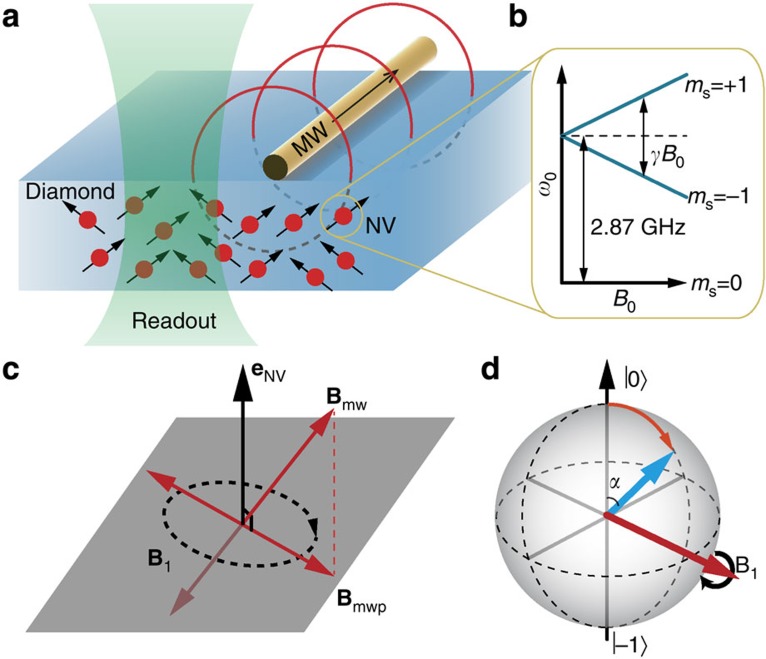
Experimental setup and principle of the MW magnetometry. (**a**) Schematic view of the setup for the demonstration of vector MW magnetometry. The 532-nm green laser is focused several micrometres below the top surface of the diamond. A copper wire of 22 μm diameter above the diamond plate is used for generating the MW field. The MW passes through the copper wire and generates a linearly polarized oscillating magnetic field (red lines). (**b**) Zeeman splitting of the electron spin of the NV centre. (**c**) Schematic view of the MW magnetic field vectors and axis of the NV centre in the laboratory frame. The dark grey surface is perpendicular to the axis of the NV centre. (**d**) Bloch sphere view of the dynamics of the spin state.

The main idea of the MW magnetometry is based on the Rabi oscillation of a solid-state spin driven under resonant MW magnetic field. The Hamiltonian of the NV’s electron spin is *H*_0_=Δ*S*_z_^2^*+γB*_0_*S*_z_+*γ***B**_mw_(*t*)·**S**, where Δ=2.87 GHz is the zero field splitting, *γ*=2.8025 MHz Gauss^−1^ is the gyromagnetic ratio of the electron spin, **S** and *S*_z_ are the associated spin operator and its z component, and **B**_mw_(*t*)=**B**_mw_·cos(2*πft)* is the MW magnetic field. We selectively address the spin operation on transition of *m*_*s*_=0↔−1 (denoted as |0› and |−1›, respectively) with a transition frequency of *ω*_0_=Δ−*γB*_0_ (Fig. 1b). Then the system can be treated similarly as spin 1/2. The resonance condition *ω*_0_*=f* can be fulfilled by adjusting *B*_0_. The component of the vector **B**_mw_ that perpendicular to *S*_z_, denoted as **B**_mwp_, can be treated as the sum of two circular polarized field with opposite rotating directions. The left-hand rotating circular polarized field *B*_1_ with an amplitude of 
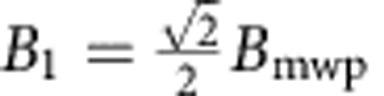
 directly drives the spin rotation (Fig. 1c). The Hamiltonian *H*_0_ can be simplified in the rotation frame as *H*_*r*_*=γB*_1_*S*_*x*_ (Fig. 1d), which results in the spin state oscillating between |0› and |−1› with Rabi frequency Ω_*r*_=*γB*_1_. In the Bloch sphere representation, the spin state vector rotates around **B**_1_ by an angle of *α=2πγB*_1_*τ* after a sensing time *τ*.

**Figure 2:**
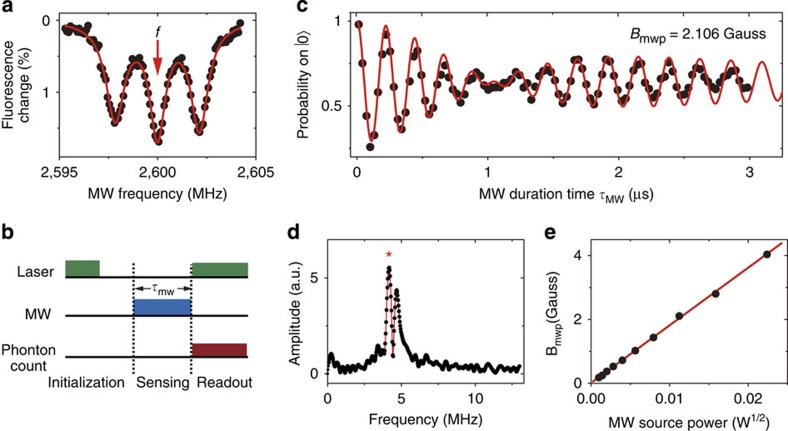
Experiment results of the MW magnetic field amplitude measurement. (**a**) Electron spin resonance lines of the NV centres. (**b**) Pulse sequence of vector MW magnetometry. The 5-μs laser pulse and 2-μs waiting time initialize the NV centre to |0›. Then the MW is on and the NV centre sense the MW magnetic field. Finally, the laser is on and the amount of photons is counted to determine the spin state. (**c**) Rabi oscillations. Each point of the data is repeated for 1.5 × 10^5^ times to increase the signal to noise ratio. The fitting yields *B*_mwp_=(2.106±0.002) × 10^−4^ T and a sensitivity of 1.0 μT Hz^−1/2^. (**d**) Fourier transformation of Rabi oscillations in **c**, with a red line connecting the data. The frequencies of the peak (*) and the other one are 4.17 MHz and 4.68 MHz. (**e**) Measured projected MW magnetic field versus MW source power.

Decoherence of the NV spin states needs to be taken into account in an actual case. After considering the interaction between NV and the ^14^N nuclear spin and the noise due to ^13^C nuclear spin bath, we have the whole Hamiltonian *H=H*_0_*+***S**·*A*·**I***+QI*_z_^2^*+bS*_z_, where **I** is the ^14^N nuclear spin operator, *A* is the hyperfine coupling tensor and *b* denotes the effective fluctuation magnetic field of the noise^23^,^24^. There are two factors need to be considered. One is the off resonance due to the hyperfine interaction with the nitrogen nuclear spin. The probability on |0› under off resonance MW driving is written as^25^


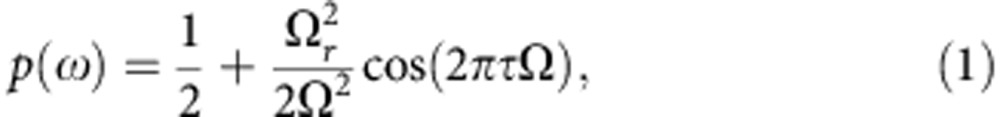


where 
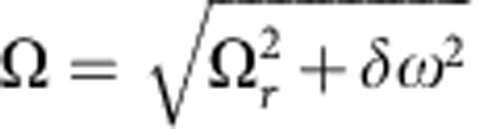
 is the Rabi frequency under off-resonance frequency detuning *δω=ω−f*. In existence of ^14^N nuclear spin, the electron spin resonance transition line splits into three peaks with frequencies *f+m*_*I*_*A*, where *m*_*I*_=0,±1 denotes the spin state of ^14^N nuclear spin (Fig. 2a). As a result, there are two frequencies Ω_fast_ and Ω_slow_ in the Rabi oscillation curve fulfilling the relation of Ω_fast_^2^=Ω_slow_^2^+*A*^2^. Ω_slow_ is the Rabi frequency of the on-resonance peak and indicates *B*_1_ value. The other factor is the inhomogeneous line broadening effect caused by the spin bath noise that dominates the signal decay. The broadened line shape can be characterized by adding a Gaussian type noise to the electron spin^23^,^24^,^25^:


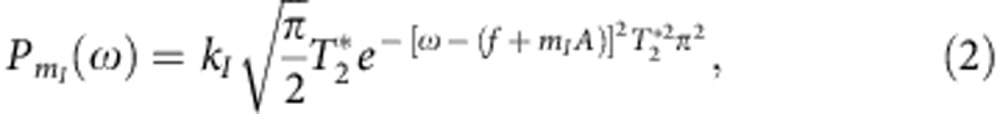


where *k*_*I*_ is the relative height of each peak and *T*_2_* is the electron spin dephasing time. The final signal can be obtained from integration of all the off-resonance contributions in the three peaks:


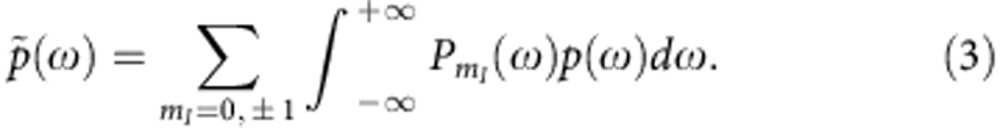


**Figure 3:**
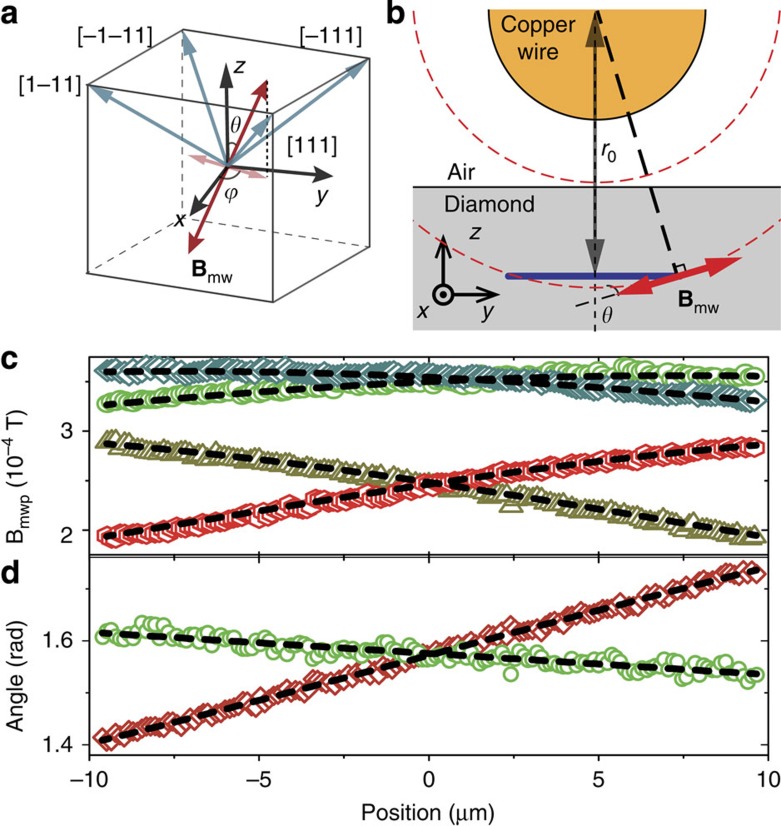
Experiment results of vector MW magnetometry. (**a**) Definition of the vector and angle. (**b**) Schematic view of the MW magnetic field distribution and the scanning line. In the near field, the oscillation current in the copper wire generates a linearly polarized magnetic field with a direction perpendicular to the copper wire and along the tangent. The coordinate is set as follows: *x* is along the copper wire, *y* is perpendicular to the copper wire and parallel to the diamond surface, and *z* is vertical to the diamond surface. The scanning line (black line) is made below the copper wire and along the *y* axis. (**c**) Measured *B*_mwp_ with the magnetic field along [111] (triangle), [–111] (diamond), [1–11] (rhombic) and [–1–11] (hexagon). The fitting curve to the data (dashed line) gives the distance *r*_0_=47.49 μm. (**d**) *θ* (diamond) and *ϕ* (circle) calculated from the data in **c**. The dashed line is the fitting curve to the data. The fitting yields the angular resolution of *δθ*=5.7 mrad and *δϕ*=9.9 mrad.

The magnetic field vector can be reconstructed by making use of NV centres with four different orientations (Fig. 3a), [111], [–111], [1–11], [–1–11] (labelled as 1, 2, 3, 4). The static magnetic field can be aligned subsequently to each of the four orientations, and then the set of NV centres of the orientation is selectively driven and its Rabi oscillation is measured to deduce the relevant projected MW magnetic field 
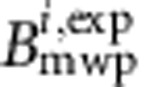
 (*i*=1, 2, 3 and 4), which is related to **B**_mwp_, as shown in Fig. 1c, by


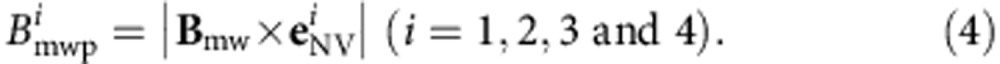


The four relations in equation (4) are over-complete but theoretically consistent in determination all the three components of **B**_mw_=(*B*_x_, *B*_y_, *B*_z_). Actually, any three of them should be sufficient, except for the exceptionally few singular points such as **B**_mwp_ being along [100], [010] or [001] (Supplementary Note 1). Hence the sensor can be a nanosized diamond with three or more appropriate NV centres. For the sample with NV centres of all the four orientations used here, we can make use of the over-completeness by applying Maximum-likelihood estimation method to the data to obtain the best estimation of the magnetic vector. The likelihood can be written as


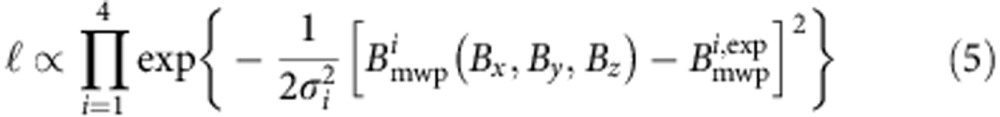


Maximizing *ℓ* will find the optimum value of the vector according to Bayes’ theorem (Supplementary Note 4). The MW magnetic field can be uniquely determined in this way.

### Experimental demonstration of the magnetometry

The probe head of the setup is shown in Fig. 1a. For the ease of demonstration, we use a chemical vapour deposition synthesized, type IIa diamond plate of 50 μm in thickness from Element Six, rather than a nanodiamond that contains NV centres as the magnetometer. The average distance between each NV centre is less than 100 nm, and there are ~20 NV centres in the laser spot. The static magnetic field **B**_0_ is aligned to the axis of each NV centre group to avoid ground-state mixing. The MW electronic circuits are designed to be 50 Ω impedance and the total power reflection is reduced to *S*_11_<−10 dB, so that a highly linearly polarized MW magnetic field is generated. As the relative permeability of diamond crystal is ~1.0000, the magnetic field inside the diamond should be the same as that outside of it. In our demonstration, we fix the applied static field to *B*_0_≈94.34 Gauss, and the corresponding frequency for the MW field is *f*=2.6000 GHz.

The pulse sequence and the measured Rabi oscillation results are shown in Fig. 2b. Figure 2c shows an example of Rabi oscillations under the MW source power of 0.13 mW. By fitting the curve, we derive the projected MW magnetic field strength. *B*_mwp_=(2.106±0.002) × 10^−4^ T (see Supplementary Note 2 and 3 for details). The fitting result is confirmed by the Fourier transformation of the data in the frequency domain (Fig. 2d). By varying the MW source power, we find that proportionality of the projected MW magnetic field strength deduced from the measured Rabi oscillation to the square root of the MW source power holds in a wide *B*_mwp_ range (Fig. 2e and Supplementary Fig. 1).

Further demonstration to show the angular resolution was performed by using the vector MW magnetometry to measure in a series of space locations the strength and orientation of the linearly polarized MW magnetic field generated by a copper wire of ~5 mm in length. The copper wire is much longer than the distance between the copper wire and the diamond surface, and so can be treated as indefinitely in length. The MW magnetic near field has a magnitude inversely proportional to the distance to the wire and an orientation along the tangent as Ampere’s circuital law. We scan the laser spot in a line to measure Rabi Oscillations. The scanning line is divided into 150 points with a step of ~130 nm and several micrometres below the diamond surface (Fig. 3b). By controlling the orientation of the static magnetic field, we measure the Rabi oscillations of the NV centres of all the four groups in each point. In prior, the diamond is put at an angle of 0.37 rad with the copper wire to avoid the singular point (Supplementary Fig. 2). The experiment results are shown in Fig. 3c (see Supplementary Table 1 for the fitting parameters). All the three components of the vector **B**_mw_ are then deduced by utilizing maximum-likelihood estimation method, and so the orientation of the vector is obtained (Supplementary Note 5). The orientation of **B**_mw_ versus position is shown in Fig. 3d, together with theoretically predicted lines. The experiment result of *ϕ* and *θ* perfectly matches the theoretical predictions of the MW magnetic field. The minimum angular resolution achieved is *δω*=5.7 mRad, which can be further improved by increasing the detecting time or photon collection efficiency^26^,^27^.

## Discussion

In conclusion, we have proposed a vector MW magnetometry scheme using NV centres in diamonds with high sensitivity and spatial resolution and under ambient conditions, and then carried out experimental demonstration of the amplitude and angular sensitivity with the measurement of the MW magnetic field vector generated by an infinitely long copper wire. In our demonstration, the spatial resolution is only diffraction limited, which gives a sensor size of ~230 nm (see Methods section). The capability of the vector MW magnetometry based on the NV centre is not limited by what we demonstrate here. By adopting wide-field imaging technology, a two-dimensional reconstruction of the MW magnetic field can be realized^28^,^29^. Based on solid-state spins of NV centre in diamond, the sensor size can be reduced to ten nanometre scale by a well-treated nanodiamond^30^. Also the working frequency can be extended to sub-terahertz band continuously by an external static field 8.6 T (ref. 31) and reach terahertz under 35 T, which is available in the current technology.

Our solid-state MW vector magnetometry will directly find its application in testing the MW electronics and materials. Owing to long-term stability of the NV centre, it can also serve for the purpose to stabilize the amplitude of the MW. Moreover, by combining the NV centres with a scanning probe microscopy, the NV centre-based scanning near-field vector MW magnetometry can be used in studying antenna radiation as a non-destructive measurement^9^. Finally, as the sensor can be downsized to nanometre scale, our scheme may promote the ferromagnetic resonance imaging^32^ to the nanoscale.

## Methods

### Experiment setup

Our setup is based on our homebuilt confocal microscopy. A temperature-stabilized diode laser (CNI MGL-III-532) is used for the initialization and readout of quantum states. The laser beam passes through a double-pass acoustic optical modulator (Crystal Technology 3200-121) to reach an isolation of about 60 dB. Then it enters a single-mode fibre acting as a spatial filter. A high numerical aperture (NA) objective lens (Olympus UPLSAPO 100XO) is used for the laser focusing and fluorescence collection. A high-resolution scanner (Asylum research) and fast steering mirror (Newport FSM-300) is used for the laser scanning. The magnetic field is applied by a permanent magnet on a three-dimensional translation stage. The fluorescence passes through a band-pass filter with 650–775 nm and 20 μm pinhole to the APD (Perkin Elemer SPCM-AQRH-14), which limit the optical detection size to be diffraction limited. The MW generated by a MW source (Agilent N5181B) is cut to square wave pulse by an MW PIN switch (CMCS0947A-C1, 3 ns rising time). An amplifier (Mini-circuits ZHL-16 W-43+) is used to amplify the MW signal.

### Thermal drift

The experimental measurements in this demonstration last for a month, so the long-term stability of the whole setup is crucial. First, we put the whole probe head in a chamber with temperature stability of ±50 mK to reduce mechanical thermal drift, which enables us to focus on the same point for a long time without any drift. Second, we put some diamond nanocrystal with NV centres on the diamond surface for locating and carrying out position feedback. Finally, the most important thing is to keep all the MW circuit stable to ±0.5 K and keep a constant output power, so that the MW magnetic field amplitude remains stable during the measurement.

### Sensor size and spatial resolution

In our demonstration, the diamond sample is rather big, and the spatial resolution is the pinhole size divided by the magnification of the objective lens, with the low limit being the Abbe diffraction limit, which can be written as:


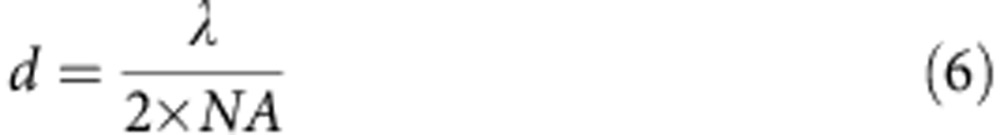


With our band-pass filter wavelength, *λ*~650 nm, we obtain *d*~230 nm. However, a well-selected nanosized diamond could be used to reach a nanoscale spatial resolution.

## Author contributions

P.W., Z.Y., F.S. prepared the setup and performed the experiments; P.W., X.R. built the microwave circuits; X.X. and M.W. designed and fabricated the microwave delivery device; J.D. supervised the setup and experiments. All authors discussed the results and participated in writing the manuscript.

## Additional information

**How to cite this article:** Wang, P. *et al*. High-resolution vector microwave magnetometry based on solid-state spins in diamond. *Nat. Commun.* 6:6631 doi: 10.1038/ncomms7631 (2015).

## Supplementary Material

Supplementary Information Supplementary Figures 1-2, Supplementary Table 1, Supplementary Notes 1-5 and Supplementary References
