# Supplementary material for: High-resolution vector microwave magnetometry based on solid-state spins in diamond
Source: Nat Commun. 2015 Mar 23;6:6631. doi: 10.1038/ncomms7631 (PMC4383011; doi:10.1038/ncomms7631)
Supplement: Supplementary Information — Supplementary Figures 1-2, Supplementary Table 1, Supplementary Notes 1-5 and Supplementary References [file ncomms7631-s1.pdf]

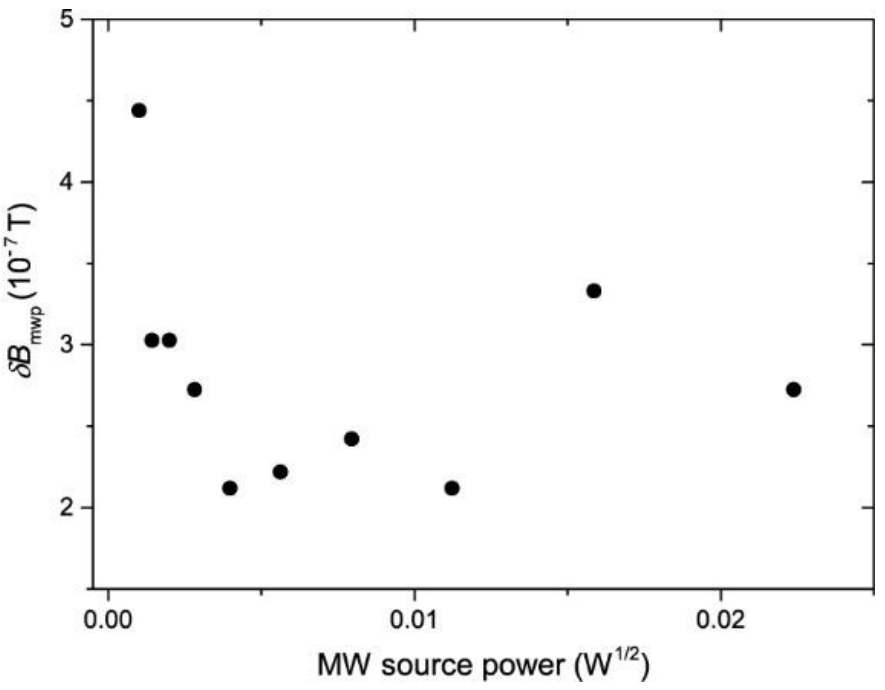

Supplementary Figure 1 | Fitting error of B_{mwp}.

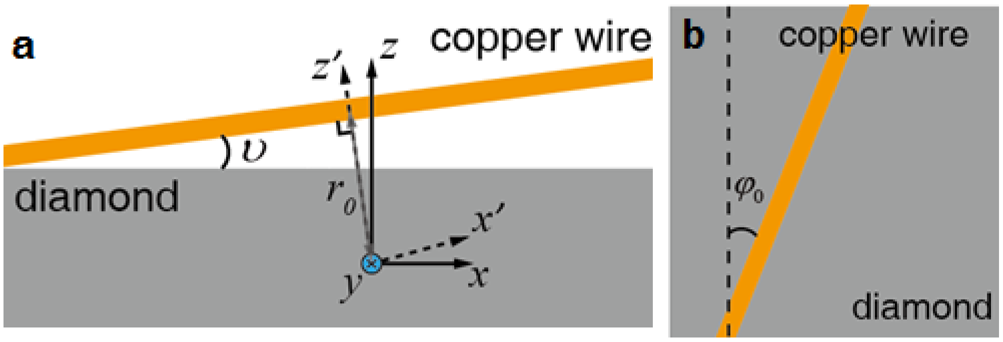

Supplementary Figure 2 | Tilt of the copper wire. (**a**) Side view**.** (**b**) Vertical view.

NV center axis	I (mA)	Standard Fitting Error of B^{i}_{mwp} (10^{-4} T)
1	145.7	0.093
2	145.0	0.093
3	145.6	0.14
4	147.4	0.065

Supplementary Table 1 | Fitting parameter of B^{i}_{mwp} **.**

Supplementary Note 1 Solution analysis of equations (4)

Equation (4) is unfolded to:

$$\begin{cases} B_x^2 + B_y^2 + B_z^2 - B_x B_y - B_x B_z - B_y B_z = \frac{3}{2} B_{\mathrm{mwp}}^{[111]2} \\ B_x^2 + B_y^2 + B_z^2 + B_x B_y + B_x B_z - B_y B_z = \frac{3}{2} B_{\mathrm{mwp}}^{[-111]2} \\ B_x^2 + B_y^2 + B_z^2 + B_x B_y - B_x B_z + B_y B_z = \frac{3}{2} B_{\mathrm{mwp}}^{[1-11]2} \\ B_x^2 + B_y^2 + B_z^2 - B_x B_y + B_x B_z + B_y B_z = \frac{3}{2} B_{\mathrm{mwp}}^{[-1-11]2} \end{cases} . \quad (1)$$

We can get a solution by using all the four equations in (Supplementary Equation 1):

$$\begin{cases} B_x = K\sqrt{\frac{L}{MK}} \\ B_y = \sqrt{\frac{KM}{L}} \\ B_z = M\sqrt{\frac{L}{MK}} \end{cases} , \quad (2)$$

Where

$$\begin{aligned} K &= \frac{3}{2}\left(-B_{\mathrm{mwp}}^{[111]2} + B_{\mathrm{mwp}}^{[-111]2} + B_{\mathrm{mwp}}^{[1-11]2} - B_{\mathrm{mwp}}^{[-1-11]2}\right) \\ L &= \frac{3}{2}\left(-B_{\mathrm{mwp}}^{[111]2} + B_{\mathrm{mwp}}^{[-111]2} - B_{\mathrm{mwp}}^{[1-11]2} + B_{\mathrm{mwp}}^{[-1-11]2}\right) \\ M &= \frac{3}{2}\left(-B_{\mathrm{mwp}}^{[111]2} - B_{\mathrm{mwp}}^{[-111]2} + B_{\mathrm{mwp}}^{[1-11]2} + B_{\mathrm{mwp}}^{[-1-11]2}\right) \end{aligned} . \quad (3)$$

The solution (S2) can determine the unique solution of equations (4). The singular point occurs when $KML = 0$, corresponding to $B_x = B_y = 0$, $B_x = B_z = 0$ or $B_y = B_z = 0$.

Supplementary Note 2. Fit to the Rabi Oscillations

We assume that the spin bath noise b is a static noise in the experiment. The static perturbation can be approximated as a magnetic field with a Gaussian distribution which results in a Gaussian type line broadening in the form of equation (2) in the Main text. The strength of b can be characterized by the dephasing time $\tilde{b} = 1\big/\sqrt{2}T_2^*$.

To fit the experiment result of Rabi Oscillations, we make some approximation on equation (3) for it is not suitable for the least squares fit. In our diamond sample, T_2^* of the different NV centers is in range of 0.5 to 1 μs, so $\tilde{b}$ is in the range of 0.7 to 1.4 MHz, which is smaller than the hyperfine splitting A.

For the case of $\omega_r > \tilde{b}$ and ω_r □ $\tilde{b}$, we take the result from Supplementary Ref. 1:

$$P_0(t)_{I=0} = a + b\left(1+\beta^2\right)^{-0.25}\cos\left(2\pi\Omega t + \frac{1}{2}\arctan\beta\right), \tag{4}$$

$$\begin{aligned} P_0(t)_{I=\pm1} = a + \left(\frac{1-a-\kappa b}{1-\kappa}\right)\left(1+\beta^2\right)^{-0.25} e^{-A^2T_2^{*2}\beta/(1+\beta)} \\ \cos\left[2\pi\Omega t + \frac{1}{2}\arctan(\beta) + A_0^2T_2^{*2}\beta\left(2-\beta^2\right)/\left(1+\beta^2\right)\right], \end{aligned} \tag{5}$$

where $\beta = \dfrac{4t}{\pi\Omega T_2^{*2}}$, a and b is the fitting parameter corresponding to the total transition rate of $|0\rangle \leftrightarrow |-1\rangle$.

For the case of ω_r □ $\tilde{b}$, only the on-resonance transition is considered and the other off-resonance transition can be neglected. Then the Rabi oscillation curve is a cosine curve with an exponential decay. This curve is fitted by sine damping. The fitting error is shown in Supplementary Figure 1.

Supplementary Note 3 Sensitivity

From the fitting to the nutation, we get the fitting error δB_{mwp} of the projected MW magnetic field. The sensitivity is given by:

$$\eta = \frac{\sqrt{nT}}{\delta B_{\mathrm{mwp}}} \quad , \tag{6}$$

where n is the average number of experiment and T is the total sensing time in a Rabi Oscillations Curve. From the fitting data, we get a minimum sensitivity of 1.0 μT $\mathrm{Hz}^{-1/2}$.

Supplementary Note 4 Maximum-likelihood estimation

When there are experiment errors, there is a chance of $KM/L < 0$ and then the experiment fails. In this case, the Supplementary Equation 3 cannot be used any more. We assume that the experiment result is B_{mwp}^{i} with a standard deviation $\delta B_{\text{mwp}}^{i}$. The experiment result follows the Gaussian distribution, which can be written as

$$F\left(B_{\text{mwp}}^{i} \,\middle|\, B_{\text{mwp}}^{i}\left(B_x, B_y, B_z\right)\right) = \frac{1}{\delta B_{\text{mwp}}^{i}\sqrt{2\pi}} \exp\left\{-\frac{\left(B_{\text{mwp}}^{i}{}' - B_{\text{mwp}}^{i}\right)^2}{2\left(\delta B_{\text{mwp}}^{i}\right)^2}\right\} \tag{7}$$

The probability of measuring each projected MW magnetic field is independent so the joint density function is

$$\ell = F\left(\left\{B_{\text{mwp}}^{i}\right\} \middle| \mathbf{B}_{\text{mw}}{}'\right) = \prod_{i=1}^{4} F\left(B_{\text{mwp}}^{i} \middle| B_{\text{mwp}}^{i}{}'\right) \propto \exp\left\{-\zeta\right\} \tag{8}$$

With

$$\zeta = \sum_{i}\left(B_{\text{mwp}}^{i}{}' - B_{\text{mwp}}^{i}\right)^2 \tag{9}$$

According to Bayes' theorem, for the randomly distributed B_{mwp}^{i} and $\mathbf{B}_{\text{mw}}$, maximizing the value of Supplementary equation 8 produces the optimum value of $\mathbf{B}_{\text{mw}}$ for the given measurement. This corresponds to minimize ζ.

Supplementary Note 5 Experimental data analysis

Because of the imperfection of the setup, there is a tilt between the copper wire and the diamond surface. This tilt needs to be corrected in data analysis. Assume that the angle between the copper wire and the diamond is υ (Supplementary Figure 2). We build two set of coordinates, with the same *y*-axis. Then the MW magnetic field vector in $x'yz'$ coordinate is written as:

$$B'_x = 0,\ B'_y = B_{\mathrm{mw}} \cdot \cos\left(\mathrm{acrtan}\frac{y}{r_0}\right),\ B'_z = B_{\mathrm{mw}} \cdot \sin\left(\mathrm{acrtan}\frac{y}{r_0}\right) \tag{10}$$

After the transformation of coordinates to xyz, it is

$$B_x = B'_z \cdot \sin\upsilon,\ B_y = B'_y,\ B_z = B'_z \cdot \cos\upsilon \tag{11}$$

Then

$$\begin{aligned} \theta &= \arctan\frac{B_z}{\sqrt{B_x^2 + B_y^2}} + \frac{\pi}{2} \\ &= \arctan\frac{\cos\upsilon \sin\left(\arctan\frac{y}{r_0}\right)}{\sqrt{\sin^2\left(\arctan\frac{y}{r_0}\right)\sin^2(\upsilon) + \cos^2\left(\arctan\frac{y}{r_0}\right)}} + \frac{\pi}{2}\ , \\ \varphi &= \arctan\frac{B_y}{B_x} + \chi_{[0,\infty)}(y)\times\pi = \arctan\frac{r_0}{y\sin\upsilon} + \chi_{[0,\infty)}(y)\times\pi \end{aligned} \tag{12}$$

where $\chi_{[0,\infty)}(y)$ is the indicator function with $\chi = 1$ when $y \geq 0$ and $\chi = 0$ when $y \leq 0$. This is in good agreement with the experiment result (Fig. 3 in the main text) with $\upsilon = 0.1994$ rad and $r_0 = 47.49$ μm obtained by least square fitting.

Then we use the obtained υ and r_0 to fit each set of B_{mwp} independently From Ampere's law, the amplitude of MW magnetic field is

$$B_{\mathrm{mw}} = \frac{\mu I}{2\pi\sqrt{r_0^2 + y^2}} \tag{13}$$

Where μ is the permeability of diamond, *I* is the MW current amplitude.

$$B_{\mathrm{mwp}}^{[111]} = \frac{\mu I \cos\nu}{\sqrt{6}\pi(y^2 + r_0{}^2)}\sqrt{\frac{y^2 + r_0{}^2}{\cos^2\nu} - U - V - W}$$

$$B_{\mathrm{mwp}}^{[-111]} = \frac{\mu I \cos\nu}{\sqrt{6}\pi(y^2 + r_0{}^2)}\sqrt{\frac{y^2 + r_0{}^2}{\cos^2\nu} + U - V + W}$$

$$B_{\mathrm{mwp}}^{[1-11]} = \frac{\mu I \cos\nu}{\sqrt{6}\pi(y^2 + r_0{}^2)}\sqrt{\frac{y^2 + r_0{}^2}{\cos^2\nu} + U + V - W}$$

$$B_{\mathrm{mwp}}^{[-1-11]} = \frac{\mu I \cos\nu}{\sqrt{6}\pi(y^2 + r_0{}^2)}\sqrt{\frac{y^2 + r_0{}^2}{\cos^2\nu} - U + V + W}$$

With all the parameters:

$$U = (r_0\cos\nu)^2\sin\varphi_0\cos\varphi_0 + ((r_0\sin\nu)^2\sin\varphi_0\cos\varphi_0 - y^2\sin\varphi_0\cos\varphi_0 - r_0 y\sin\nu\cos 2\varphi_0)\tan^2\nu$$
$$+ r_0\sin\nu(r_0\sin\nu\sin 2\varphi_0 - y\cos 2\varphi_0)$$

$$V = y(-r_0\cos\nu\cos\varphi_0 - \tan\nu(r_0\sin\nu\cos\varphi_0 + y\sin\varphi_0))$$

$$W = y(-r_0\cos\nu\sin\varphi_0 - \tan\nu(r_0\sin\nu\sin\varphi_0 - y\cos\varphi_0))$$

Where $\varphi_0 = 0.37\ \mathrm{rad}$ is the transverse angle between the diamond and the copper wire. The fitting result by treating *I* as a parameter is shown in Supplementary Table 1. We note that the four values obtained from the 4 sets of experimental data are consistent with each other, considering that there are experimental and model errors.

Supplementary References:

1. Hanson, R., Dobrovitski, V. V., Feiguin, A. E., Gywat, O. & Awschalom, D. D. Coherent dynamics of a single spin interacting with an adjustable spin bath. *Science* **320**, 352-355, doi:DOI 10.1126/science.1155400 (2008).
